# Medicaid Expansion Increases Treatment for Patients with Colon Cancer

**DOI:** 10.3390/cancers17020207

**Published:** 2025-01-10

**Authors:** John Morgan Lyons, Denise M. Danos, Lauren S. Maniscalco, Yong Yi, Omeed Moaven, Xiaocheng Wu, Quyen Chu

**Affiliations:** 1 Department of Surgery, Louisiana State University Health Sciences Center, 2021 Perdido Street, 8th Floor, New Orleans, LA 70112, USA; ddanos@lsuhsc.edu (D.M.D.); omoaven@lsuhsc.edu (O.M.); 2 Our Lady of the Lake Regional Medical Center, 7777 Hennessy Blvd, Baton Rouge, LA 70808, USA; 3 School of Public Health, Louisiana State University Health Sciences Center, 2020 Gravier Street, 3rd Floor, New Orleans, LA 70112, USA; lspiza@lsuhsc.edu (L.S.M.);; 4 College of Medicine, Howard University, 2041 Georgia Ave NW Rm. 4B-16, Washington, DC 20019, USA; 5 Division of Surgical Oncology, The Roy L. Schneider Endowment, Washington, DC 20019, USA

**Keywords:** Affordable Care Act, Medicaid expansion, health insurance, health disparities, colon cancer treatment

## Abstract

Introduction: Medicaid expansion (ME) has positively impacted colon cancer screening. ME’s effect on colon cancer treatment is less clear. This study analyses the effect of ME on patterns of colon cancer treatment. Methods: Patients with primary invasive colon cancer were identified using the Louisiana Tumor Registry. Patients diagnosed with colon cancer prior to ME (2014–2015) were compared to those diagnosed after (2017–2018). Coordinate variables were analyzed using Fisher’s exact test. Treatment status was modeled with multivariable logistic regression and the results are reported as adjusted odds ratios. Results: The proportion of uninsured patients decreased following ME (5.5 versus 1.9, *p <* 0.001), with the greatest reductions among patients between 45 and 54 years old (13.5% to 3.5%, *p <* 0.0001), African Americans (8.9 to 2.1%, *p* < 0.0001), and those in high-poverty neighborhoods (7.1 to 2.1%, *p* < 0.0001). Following ME, all patients with Stage I-III disease were more likely to receive surgery (OR = 1.95; 95%: CI 1.21–3.14)—especially the extremely impoverished (OR = 2.39; 95% CI 1.41–4.02). Young patients with Stage IV colon cancer were more likely to receive chemotherapy (OR-1.6; 95% CI 1.03–2.4). Patients with Stage IV colon cancer were less likely to receive treatment within 30 days of diagnosis (OR = 0.7; 95% CI 0.5–0.9), but, on subset analysis, this was only observed in non-Medicaid patients. Conclusion: ME is associated with increased treatment for patients with colon cancer, and it did not appear to affect time to treatment. However, it seems to affect different subsets of the population differently.

## Simple Summary

Medicaid expansion remains a controversial topic—especially in the deep south. While expansion has been associated with both increased screening and earlier stage at diagnosis we studied treatment patterns among patients diagnosed with colon cancer in the 2 years prior to and the 2 years after Medicaid expansion. We found increased rates of surgery and chemotherapy among several vulnerable subsets of the population. To our knowledge, this is the first study to demonstrate the positive effect of expansion on colon cancer treatment, and it is especially unique not only because the results were noted within 2 years of the expansion, but also because it highlights results from Louisiana—the only deep south state in the US to enact Medicaid expansion to date.

## 1. Introduction

The Affordable Care Act, passed in 2010, provided states a historic opportunity to expand Medicaid coverage to childless adults with incomes at or below 138 percent of the federal poverty level [1]. This Medicaid expansion (ME) has lowered the uninsured rate in expansion states among low-income, non-elderly adults by 17 percentage points—from 35% to 17%—between 2013 and 2019. By comparison, the rate in non-expansion states has dropped only 9 points—from 43% to 34%—during the same period [2]. Several expansion states have consistently demonstrated net healthcare savings as the ME provision defers more of the state-level healthcare costs to the federal government [3,4].

However, concerns exist that ME discourages private employers from offering commercial insurance, relegating employees to a less comprehensive product with inferior coverage that will be funded by taxpayers [5,6,7]. In addition, several large studies, including one randomized trial, have failed to show improved healthcare outcomes in Medicaid recipients when compared to those without insurance [6,7]. Thus, ME remains controversial and has not been universally accepted. For these reasons, ME warrants independent, non-political scientific analysis, and it is this research that should be used to guide national healthcare reforms and funding decisions.

The impact of ME on colon cancer screening has been studied by several researchers. These studies, in addition to a systemic review, have shown conflicting results regarding the efficacy of ME on colon cancer screening [8]. Health insurance has been shown to be a major driver of colon cancer treatment [9], and treatment patterns may be a better outcome to assess the effect of insurance. Treatment seems to be less affected by patient preference, education, and public health campaigns than screening [10]. Because of this, changes in treatment patterns could occur more quickly than those seen in screening. To date, however, only one investigator, using the national cancer database (NCDB), has studied the impact of ME on colon cancer treatment [11]. While they noted increases in treatment within integrated network programs, the overall receipt of either surgery or chemotherapy was unaffected by ME.

While the NCDB captures data exclusively from Commission on Cancer-accredited cancer centers, we hypothesized that a treatment effect may be more evident using a more heterogeneous dataset representing a broader cross-section of backgrounds. Therefore, we chose to study the effect of Medicaid expansion on colon cancer using data from the state of Louisiana. To date, 41 states, including the District of Columbia, have expanded their Medicaid coverage, and nine of the ten non-expansion states are in the deep south [12]. Louisiana is the only deep south state to enact traditional Medicaid expansion [13]. This provides an opportunity to observe unique outcomes within the bayou state that may be otherwise diluted within the noise of larger national databases. Additionally, as of 2019, Louisiana ranked no two in colon cancer incidence and no four in colon cancer mortality in the United States [14,15]; and in 2015, Louisiana’s uninsured rate was among the top ten worst in the United States [9].

This study aims to evaluate the demographics, stage distribution, and treatment patterns of patients diagnosed with colon cancer in Louisiana before and after the enactment of Medicaid expansion.

## 2. Methods

Study data were derived from the Louisiana Tumor Registry—a statewide, population-based cancer registry funded by the National Cancer Institute’s Surveillance, Epidemiology and End Results (SEER) Program and the Centers for Disease Control and Prevention’s National Program of Cancer Registries. These data are available by request from the Louisiana Tumor Registry (New Orleans, LA, USA). The Louisiana Tumor Registry has collected, validated, and consolidated cancer incidence data for 100% of the state’s population to support cancer prevention and control research activities since the 1980s [16]. Primary invasive colon cancer cases diagnosed among those aged 20 and older were selected using the International Classification of Diseases for Oncology (ICD-O-3) topographic codes C180-C189 and histology codes 8140, 8210, 8263, 8480, 8490. The ICD-O-3 codes of the following locations were not included: C199 (rectosigmoid), C209 (rectal), C210-C218 (anal) [17]. Cases diagnosed by either autopsy or death certificate were not included in the data extract. The study was exempted from Institutional Review Board approval by the Louisiana State University Health Sciences Center, New Orleans.

Variables pertaining to demographics and patient characteristics were standard North American Association of Central Cancer Registries (NAACCR) items listed in the registry. American Joint Commission on Cancer (AJCC) stage was defined by pathologic stage, or clinical stage if the pathologic stage was not recorded. Primary payer/insurance carrier at the time of initial diagnosis and/or treatment at the reporting facility was classified according to the following NAACCR codes: 1–2 (No insurance/self-pay); 20–21 (Private Insurance); 31–35 (Medicaid); 99 (unknown). Cases with codes 10 and 65–68 (Medicare, Tricare, Military, Veterans Affairs, or not otherwise specified) were classified as “other”. Cancer treatment status was determined using the NAACCR summary codes for the surgical removal of the primary malignancy (Item 1294), treatment of the regional lymph nodes at the time of surgery (Item 1292), and treatment with single- or multi-agent chemotherapy (Item 1390). Cases with in situ disease (Stage 0 at diagnosis; n = 275), or those missing information on lymphadenectomy (n = 33) were excluded from the study dataset.

Residential rural–urban status was based on the address at diagnosis and classified according to the Census Bureau’s urban–rural indicator code as captured in 2010 [18,19]. Codes for neighborhood poverty level were based on the address at diagnosis and organized according to the American Community Survey, which classifies neighborhoods according to the percentage of inhabitants below the federal poverty level in the following tiers: 0–<5%, 5–<10%, 10–<20%, 20–100%. The primary outcome was the rate of colon cancer treatment (surgery, lymphadenectomy, and chemotherapy) following ME. Secondary outcomes were changes in the uninsurance rate among colon cancer patients following ME and time from diagnosis to treatment. Cases without valid time to treatment data were excluded from the time to treatment analyses (n = 119).

### Statistical Analysis

ME was enacted in Louisiana in July 2016. A detailed case comparison was completed for two years before ME (2014–2015) and two years after ME (2017–2018). Cases diagnosed in the year 2016 were excluded. Changes in demographic, staging, treatment, and outcomes between these two periods were analyzed using Chi-squared tests. Time to treatment was compared with the log-rank test, where cases with no treatment were censored at the time of last follow-up. Rates of uninsured patients in subpopulations were compared using Fisher’s exact tests.

Treatment outcomes (surgery, lymphadenectomy, chemotherapy, treatment within 30 days) were modeled with logistic regression, controlling for age, sex, and race as fixed effects. The models included an interaction effect between ME and stage, and the effect of ME within stage groups was estimated using linear contrast specifications. Cases with unknown chemotherapy status were excluded from the chemotherapy models. Estimates of time to treatment were calculated using the Kaplan–Meier method and compared using log-rank tests. Statistical analyses were executed in SAS/STAT software, version 9.4 (Cary, NC, USA). All statistical tests were 2-sided, with a *p*-value < 0.05 used to identify statistical significance.

## 3. Results

### 3.1. Characteristics of the Cohort

A total of 5462 patients diagnosed with colon cancer were included in the study. Table 1 provides a summary of the overall study cohort. The mean age at diagnosis was 66 years. Males comprised 50.6% of the cohort, and 65.5% were white, which is similar to the demographic distribution reported in the US Census for Louisiana (69%) [20]. Most patients resided in an urban setting (73.1%), and most (63.6%) had government insurance (Medicaid or Medicare). Additionally, 80.2% of patients resided in a neighborhood where at least 10% of the residents were below the federal poverty line. The distribution of AJCC stage at diagnosis was relatively even, with 49.4% with Stage I or II disease and 50.6% presenting with Stage III or IV disease.

### 3.2. Impact of Medicaid Expansion on the Demographics of Patients with Colon Cancer

There were 2544 patients (46.6%) diagnosed with colon cancer pre-ME, and 2918 patients (53.4%) diagnosed post-ME. The demographics of colon cancer patients diagnosed pre- and post-ME did not significantly change, except that a higher proportion of patients came from impoverished neighborhoods following ME (patients from a neighborhood where >10% were below the federal poverty level—79.4% versus 81.0%, *p* = 0.0273). The AJCC-stage distribution of patients diagnosed was similar pre- and post-ME (*p* = 0.1261).

### 3.3. Impact of Medicaid Expansion on Uninsured Patients

Uninsured/self-pay patients comprised 3.6% of the study cohort. The characteristics of patients by insurance status are presented in Table 2. When compared to patients with insurance, a higher proportion of uninsured patients were African American (50.5 vs. 32.6%; *p* < 0.0001). A higher percentage of uninsured patients were diagnosed with Stage IV disease (33.2 vs. 24.4%; *p* = 0.0113). Uninsured patients were also less likely to have lymphadenectomy (74.7 vs. 8.0%; *p* = 0.0031) and more likely to have chemotherapy (45.3 vs. 35.7%; *p* = 0.0111).

Changes in the uninsured rate of all colon cancer patients before and after ME are outlined in Table 3. The uninsured rate decreased among the entire cohort of colon cancer patients post-ME (5.3 versus 1.9; *p* < 0.0001). The greatest reduction in the rate of uninsured patients were seen among the following demographics: (1) young people (6.2% decrease among 35–44 year olds, 10% decrease among 45–54 year olds, and 7.2% decrease among 55–64 year olds; *p* < 0.05), (2) black people (6.9% decrease; *p* < 0.0001), and (3) patients from more impoverished neighborhoods (3.8% decrease among those from a neighborhood with 10% or more residents living in poverty; *p* < 0.0001).

### 3.4. Impact of Medicaid Expansion on Colon Cancer Treatment

Treatment patterns, pre- and post-ME, were analyzed within the entire cohort, and they were also analyzed within the groups most affected by ME (i.e., young patients, black patients, and more impoverished patients). Treatment patterns were further broken down by stage (locoregional—AJCC Stage I—III; metastatic—AJCC Stage IV). The adjusted odds ratios from models controlling for age, sex, and race are provided in Table 4. When analyzing the entire cohort, patients with locoregional colon cancer had a 90% (*p* = 0.0063) increase in the odds of surgery on their primary and a 40% (*p* = 0.0036) increase in the odds of lymphadenectomy post-ME. Receipt of chemotherapy amongst locoregional patients was not different pre- and post-ME (*p* = 0.9624), nor was the time to treatment of these patients (*p* = 0.2518). When analyzing the entire cohort, patients with metastatic colon cancer experienced no difference in surgery (*p* = 0.6286), lymphadenectomy (*p* = 0.7222), or receipt of chemotherapy pre- and post-ME (*p* = 0.2508). However, the odds of having treatment initiated within 30 days of diagnosis were 30% less after ME (*p* = 0.0126).

When analyzing black patients with colon cancer exclusively, there was no difference in surgery, lymphadenectomy, receipt of chemotherapy, or having treatment within 30 days pre- and post-ME, regardless of stage (*p* > 0.05). When analyzing high-poverty patients, those with locoregional colon cancer were 2.4 times more likely to receive surgery on their primary (OR = 2.4; 95% CI 1.4–4.0; *p* = 0.0012) and 60% more likely to have a lymphadenectomy post-ME (OR = 1.6; 95% CI 1.2–2.1; *p* = 0.001). Receipt of chemotherapy among high-poverty patients was unchanged pre- and post-ME regardless of stage. No treatment differences were observed among patients with high poverty with metastatic disease. However, high-poverty patients with metastatic colon cancer diagnosed after ME were 40% less likely to have their treatment initiated within 30 days of diagnosis (OR = 0.6; 95% CI 0.5–0.9; *p =* 0.0078). Young patients with locoregional colon cancer experienced no difference in receipt of surgery, lymphadenectomy, or chemotherapy pre- and post-ME. However, young patients with metastatic colon cancer were 60% more likely to receive chemotherapy post-ME (OR = 1.6; 95% CI 1.0–2.4; *p =* 0.035). Moreover, young patients with metastatic colon cancer diagnosed after ME were 40% less likely to have treatment initiated within 30 days after diagnosis (OR = 0.6; 95% CI 0.4–0.9; *p =* 0.0137).

To address the concern that increases in time to treatment observed post-ME could result from cancer patients experiencing a lengthy process enrolling in Medicaid, time to treatment was analyzed stratified by insurance status. These results are outlined in Table 5. There were no changes in the time to treatment when analyzing the entire cohort *p* = 0.0746). There was no change in the time to treatment when looking exclusively at all patients with early-stage disease, but there was an increased time to treatment among all patients with Stage IV disease from 16 to 22 days (*p* = 0.0224). Stratified analyses revealed an increase in time to treatment among Medicare patients within the entire cohort (median 9 vs. 13 days, *p* = 0.0226), and among patients with metastatic disease who had private insurance (median 12 vs. 25 days, *p* = 0.0102). There were no changes in the time to treatment observed among those with no insurance, nor in those with Medicaid.

## 4. Discussion

We compared patients diagnosed with colon cancer 2 years before ME to those diagnosed 2 years after ME. We observed fewer uninsured patients diagnosed with colon cancer following ME. We noted three subgroups in which the improved uninsured rate was most pronounced: young people, people of color, and impoverished individuals (those in a neighborhood where >20% of residents were below the federal poverty level). While others have found an increase in the proportion of early-stage disease following ME [21], we did not observe significant changes in stage distribution in this study. One would expect to observe an increase in early-stage disease only if there was a corresponding increase in the colon cancer screening rate, and insurance coverage has not consistently resulted in an increase in preventative health utilization [22]. Cancer screening following ME has been studied by many others with variable results. Some have witnessed an improvement in screening rates [21], and some have observed no change [22]. The Louisiana behavior risk factor surveillance system showed no significant difference in the colorectal screening rate between 2016 and 2018 [23]; this most likely explains why, in this study, we did not observe an improvement in early-stage disease.

In the current study, we found that certain groups of patients experienced a longer time from diagnosis to treatment following ME. We hypothesized that this was due to a higher volume of newly insured cancer patients overwhelming an already strained oncology workforce. This has been described in other specialties throughout healthcare both in and outside oncology [24,25]. However, this factor does not appear to have driven our results because subset analysis only demonstrated an increase in time to treatment among patients with commercial insurance and Medicare. The time to treatment among Medicaid patients trended toward a decrease following ME. The increased time to treatment we observed may be less dependent on insurance status and more associated with a national trend of growing cancer prevalence within an aging population, increasing complexity of care, and a shrinking provider pool [26,27].

We did observe improvements in treatment following ME. Firstly, we noted an increase in the utilization of chemotherapy in young people with metastatic colon cancer. Notably, this observation was only in young patients, not in people of color nor in patients from impoverished areas. This is probably because young people naturally have fewer debilitating comorbidities [28] and less frailty [29], making them better candidates for prolonged and often palliative treatment. Thus, among those with metastatic disease, ME seems to more positively impact young people than other groups [30].

We also found that the utilization of surgery was increased in the cohort of patients with locoregional (Stage I–III) disease. These increases were observed in all three subgroups (young people, people of color, and impoverished individuals); however, statistical significance was only appreciated in those who were the most impoverished. Hoehn et al. is the only other group, to date, that have also studied colon cancer treatment patterns following ME [11]. They found that patients who underwent surgery for colon cancer following ME had fewer emergency cases and a higher rate of minimally invasive surgery—two factors associated with improved cancer outcomes. However, they did not report an overall increase in the surgical treatment of colon cancer. Therefore, our finding correlating an increase in surgery for locoregional colon cancer following ME is unique and, to our knowledge, the first of its kind.

These results indicate a very positive association between improved colon cancer treatment and ME. However, these results also highlight some very significant limitations of the ME program that should be acknowledged by providers, patients, and policymakers. Firstly, ME appears to affect different groups differently. For example, in the current study, chemotherapy use was only improved in young people with advanced-stage disease, and the use of surgery was only significantly improved in impoverished individuals. This variable effect of ME on colon cancer in different groups has been shown by others as well. Qian et al. queried the SEER database to calculate age-adjusted incidence rates of colorectal cancer pre- and post-ME [31]. They found that rates varied according to race/ethnicity, with increases seen in non-expansion states only for Hispanic and Asian or Pacific Islander patients, but not among black or non-Hispanic white patients. Gan et al. studied the effect of colon cancer screening on over 900,000 individuals in Kentucky [21]. While they observed increases in the screening rate among all individuals post-ME, they observed the biggest increases among younger individuals, males, and Appalachian residents. Additionally, the proportion of early-stage colorectal cancer (Stage I/II) increased by 9.3% for Appalachians, while there was little change for non-Appalachians (−1.5%).

Secondly, the true impact of ME on colon cancer may take many years to realize. Fedewa et al. used the Behavioral Risk Factor Surveillance System to assess the rate of colon cancer screening among very early, early, late, and non-expansion states [32]. They noted that the past-2-year screening rate increased by 8.0% in very early expanders and only 2.8% in non-expansion states. However, states expanding Medicaid after the “very early” states showed screening rates that were no different than those of non-expansion states. Other experts have also acknowledged that 2 years may be a very short interval in which to see improvements in population-level health status [25], and large-scale improvements in cancer outcomes may take several years following expansion to be appreciated.

Thirdly, while ME remains a major factor in healthcare access, there are many other relevant barriers that exist independent of insurance coverage. Financial barriers—such as out-of-pocket expenses, co-pays, deductibles, and time away from work—have all been shown to limit healthcare access [33]. Countless other non-financial barriers—cultural, language, psychological and emotional, socioeconomic, transportation, etc.—play a huge role in access to care regardless of insurance status [34,35,36]. Moreover, while ME has increased the number of patients with Medicaid, the Medicaid-provider pool in the colon cancer space has not increased accordingly, which can render it more difficult for these individuals to access care [37].

These barriers are real, but many of them are difficult to quantify, and this makes them impossible to control for in a research setting, which can confound our results; that is a limitation of this study. The retrospective nature of this registry study also presents potential limitations, including the implicit biases of both treatment and patient selection. This is an observational study, capable of describing associations between variables, but unable to prove causality. Additional limitations include the potential for the incomplete reporting of some variables as well as the potential for observational and/or confirmation bias. However, we utilized a large heterogeneous dataset comprising patients from both rural and urban settings in both community and academic practices from a single deep south state that ranked amongst the highest in uninsured individuals pre-ME.

## 5. Conclusions

We demonstrated increased treatment of colon cancer following Medicaid expansion, primarily among the young and the most-impoverished individuals. We observed longer waiting times for treatments, but these did not appear to be directly driven by ME. We did not observe an increase in early-stage disease, underscoring the prevalence of other barriers to healthcare and emphasizing the need to continue efforts to reduce these barriers among vulnerable populations. Medicaid expansion remains a controversial political topic, and several US states have not expanded Medicaid services because of this. This study broadens the understanding of how insurance coverage affects cancer care in the US. However, the positive results associated with ME outlined in this manuscript (and described by others) should be considered by policymakers regarding healthcare reforms.

## Figures and Tables

**Table 1:** Case characteristics for colon cancer in Louisiana before and after Medicaid expansion.

	All	Before Expansion (2014–2015)	After Expansion (2017–2018)	*p*-Value
All, n (%)	5462 (100.0)	2544 (100.0)	2918 (100.0)	
Age at Diagnosis, n (%)				0.5862
Less than 45	302 (5.5)	142 (5.6)	160 (5.5)	
45–54	702 (12.9)	326 (12.8)	376 (12.9)	
55–64	1322 (24.2)	639 (25.1)	683 (23.4)	
65–74	1657 (30.3)	750 (29.5)	907 (31.1)	
75 and older	1479 (27.1)	687 (27.0)	792 (27.1)	
Sex, n (%)				0.2614
Male	2766 (50.6)	1309 (51.5)	1457 (49.9)	
Female	2696 (49.4)	1235 (48.5)	1461 (50.1)	
Race, n (%)				0.5177
White	3579 (65.5)	1665 (65.4)	1914 (65.6)	
Black	1813 (33.2)	851 (33.5)	962 (33.0)	
Other	70 (1.3)	28 (1.1)	42 (1.4)	
Insurance, n (%)				<0.0001
Not Insured	190 (3.5)	134 (5.3)	56 (1.9)	
Private	1408 (25.8)	683 (26.8)	725 (24.8)	
Medicaid	480 (8.8)	153 (6.0)	327 (11.2)	
Medicare	2995 (54.8)	1373 (54.0)	1622 (55.6)	
Other	389 (7.1)	201 (7.9)	188 (6.4)	
Area Poverty, n (%)				0.1345
Less than 10%	1080 (19.8)	525 (20.6)	555 (19.0)	
10% or more	4382 (80.2)	2019 (79.4)	2363 (81.0)	
Residence, n (%)				0.1557
Rural	1469 (26.9)	661 (26.0)	808 (27.7)	
Urban	3993 (73.1)	1883 (74.0)	2110 (72.3)	
AJCC Stage, n (%)				0.1261
1	1215 (22.2)	571 (22.4)	644 (22.1)	
2	1485 (27.2)	695 (27.3)	790 (27.1)	
3	1414 (25.9)	685 (26.9)	729 (25.0)	
4	1348 (24.7)	593 (23.3)	755 (25.9)	
Surgery, n (%)				0.4946
No	703 (12.9)	319 (12.5)	384 (13.2)	
Yes	4759 (87.1)	2225 (87.5)	2534 (86.8)	
Lymphadenectomy, n (%)				0.6747
No	945 (17.3)	446 (17.5)	499 (17.1)	
Yes	4517 (82.7)	2098 (82.5)	2419 (82.9)	
Chemotherapy, n (%) ^a^				0.5638
No	2842 (52.0)	1313 (51.6)	1529 (52.4)	
Yes	1968 (36.0)	890 (35.0)	1078 (36.9)	
Unknown	652 (11.9)	341 (13.4)	311 (10.7)	

^a^ Statistical test only for cases with known status. AJCC = American Joint Commission on Cancer.

**Table 2:** Rates of uninsured colon cancer patients in Louisiana.

	Insured	Uninsured (Self-Pay)	*p*-Value
All, n (%)	5272 (100.0)	190 (100.0)	
Age at Diagnosis, n (%)			<0.0001
Less than 45	280 (5.3)	22 (11.6)	
45–54	645 (12.2)	57 (30.0)	
55–64	1231 (23.3)	91 (47.9)	
65–74	1644 (31.2)	13 (6.8)	
75 and older	1472 (27.9)	7 (3.7)	
Sex, n (%)			0.6346
Male	2673 (50.7)	93 (48.9)	
Female	2599 (49.3)	97 (51.1)	
Race, n (%)			<0.0001
White	3489 (66.2)	90 (47.4)	
Black	1717 (32.6)	96 (50.5)	
Other	66 (1.3)	4 (2.1)	
Area Poverty, n (%)			0.1605
Less than 10%	1050 (19.9)	30 (15.8)	
10% or more	4222 (80.1)	160 (84.2)	
Residence, n (%)			0.8546
Rural	1419 (26.9)	50 (26.3)	
Urban	3853 (73.1)	140 (73.7)	
AJCC Stage, n (%)			0.0113
1	1186 (22.5)	29 (15.3)	
2	1430 (27.1)	55 (28.9)	
3	1371 (26.0)	43 (22.6)	
4	1285 (24.4)	63 (33.2)	
Surgery, n (%)			0.0595
No	670 (12.7)	33 (17.4)	
Yes	4602 (87.3)	157 (82.6)	
Lymphadenectomy, n (%)			0.0031
No	897 (17.0)	48 (25.3)	
Yes	4375 (83.0)	142 (74.7)	
Chemotherapy, n (%)			0.0111
No	2757 (52.3)	85 (44.7)	
Yes	1882 (35.7)	86 (45.3)	
Unknown	633 (12.0)	19 (10.0)	

Cell counts less than 11 are suppressed for patient privacy. AJCC = American Joint Commission on Cancer.

**Table 3:** Change in the rate of uninsured colon cancer patients in Louisiana before and after Medicaid expansion.

	Decrease the Rate of Uninsured	*p*-Value
All, n (%)	3.4	<0.0001
Age at Diagnosis, n (%)		
Less than 45	6.2	0.0389
45–54	10.0	<0.0001
55–64	7.3	<0.0001
65–74	−0.9	0.0298
75 and older	0.5	0.1841
Sex, n (%)		
Male	3.1	<0.0001
Female	3.7	<0.0001
Race, n (%)		
White	1.7	0.0012
Black	6.9	<0.0001
Other	−3.6	0.5283
Area Poverty, n (%)		
Less than 10%	1.6	0.1018
10% or more	3.8	<0.0001
Residence, n (%)		
Rural	4.3	<0.0001
Urban	3.0	<0.0001
AJCC Stage, n (%)		
1	0.8	0.3719
2	4.4	<0.0001
3	2.9	0.0016
4	5.2	<0.0001

AJCC = American Joint Commission on Cancer.

**Table 4:** Estimated effects of Medicaid expansion on treatment patterns for colon cancer in Louisiana. Estimates are odds ratios and 95% confidence intervals from logistic regression models of treatment status.

	Full Cohort		Black		High Poverty		Younger than 65	
Stage I–III	OR (95% CI)	*p*-Value	OR (95% CI)	*p*-Value	OR (95% CI)	*p*-Value	OR (95% CI)	*p*-Value
Surgery	1.9 (1.2–3.1)	0.0063	2.0 (0.9–4.4)	0.0745	2.4 (1.4–4.0)	0.0012	1.9 (0.8–4.5)	0.1612
Lymphadenectomy	1.4 (1.1–1.8)	0.0036	1.4 (0.9–2.1)	0.1304	1.6 (1.2–2.1)	0.0010	1.2 (0.9–1.8)	0.2577
Chemotherapy	1.0 (0.9–1.2)	0.9624	1.1 (0.9–1.4)	0.4642	1.0 (0.9–1.2)	0.9771	1.0 (0.8–1.2)	0.9493
Treatment within 30d	0.9 (0.8–1.1)	0.2518	1.0 (0.8–1.3)	0.9954	0.9 (0.8–1.1)	0.5384	1.0 (0.8–1.3)	0.9567
Stage IV								
Surgery	0.9 (0.8–1.2)	0.6286	0.9 (0.6–1.3)	0.5220	0.9 (0.7–1.1)	0.2270	0.9 (0.6–1.2)	0.4554
Lymphadenectomy	1.0 (0.8–1.2)	0.7222	0.9 (0.6–1.3)	0.5308	0.9 (0.7–1.1)	0.2980	0.9 (0.7–1.3)	0.6562
Chemotherapy	1.2 (0.9–1.5)	0.2508	1.0 (0.7–1.5)	0.9650	1.1 (0.8–1.4)	0.6046	1.6 (1.0–2.4)	0.0350
Treatment within 30d	0.7 (0.5–0.9)	0.0126	1.0 (0.6–1.6)	0.9536	0.6 (0.5–0.9)	0.0078	0.6 (0.4–0.9)	0.0137

**Table 5:** Median time to treatment, in days, for colon cancer patients before and after Medicaid expansion.

	All			Stage I-III			Stage IV		
	Before	After		Before	After		Before	After	
	Median (95% CI)	Median (95% CI)	*p*-Value	Median (95% CI)	Median (95% CI)	*p*-Value	Median (95% CI)	Median (95% CI)	*p*-Value
All	9 (8.10)	10 (9.12)	0.0746	7 (5.7)	6 (5.7)	0.8023	16 (13.20)	22 (19.26)	0.0224
Insurance									
Private	8 (6.11)	10 (7.12)	0.2721	7 (5.10)	6 (3.8)	0.3753	12 (7.16)	25 (17.27)	0.0102
Medicaid	6 (1.14)	3 (1.6)	0.6259	3 (0.8)	1 (0.3)	0.4521	20 (5.32)	13 (4.27)	0.8649
Medicare	9 (7.11)	13 (11.14)	0.0226	7 (5.9)	9 (7.11)	0.4728	18 (13.23)	23 (20.31)	0.0710
Not-insured	12 (4.18)	2 (0.10)	0.9842	5 (0.15)	0 (0.5)	0.8440	31 (10.44)	10 (2.77)	0.7806
Other	10.5 (6.17)	10 (2.15)	0.9832	10 (3.14)	3 (1.12)	0.8957	18 (9.38)	21 (4.41)	0.7319

## Data Availability

The original data presented in the study are openly available upon request from the corresponding author and/or directly from the Louisiana Tumor Registry.

## References

[1] Rosenbaum S. (2011). The Patient Protection and Affordable Care Act: Implications for Public Health Policy and Practice. Public Health Rep..

[2] Rubin I., Cross-Call J., Gideon L. Medicaid Expansion: Frequently Asked Questions|Center on Budget and Policy Priorities. https://www.cbpp.org/research/health/medicaid-expansion-frequently-asked-questions.

[3] Buchmueller T.C., Cliff B.Q., Levy H. (2020). The benefits of Medicaid expansion. JAMA Health Forum.

[4] Richardson J.A., Llorens J.J., Heidelberg R.L. (2018). Medicaid Expansion and Louisiana Fiscal Outcomes Medicaid Expansion and the Louisiana Economy. https://gov.louisiana.gov/assets/MedicaidExpansion/MedicaidExpansionStudy.pdf.

[5] Cutler D.M., Gruber J. (1996). Does public insurance crowd out private insurance?. Q. J. Econ..

[6] Baicker K., Taubman S.L., Allen H.L., Bernstein M., Gruber J.H., Newhouse J.P., Schneider E.C., Wright B.J., Zaslavsky A.M., Finkelstein A.N. (2013). The Oregon experiment—Effects of Medicaid on clinical outcomes. N. Engl. J. Med..

[7] LaPar D.J., Bhamidipati C.M.D., Mery C.M., Stukenborg G.J., Jones D.R., Schirmer B.D., Kron I.L., Ailawadi G. (2010). Primary payer status affects mortality for major surgical operations. Ann. Surg..

[8] Xu M.R., Kelly A.M., Kushi L.H., Reed M.E., Koh H.K., Spiegelman D. (2020). Impact of the affordable care act on colorectal cancer outcomes: A systematic review. Am. J. Prev. Med..

[9] Roetzheim R., Pal N., Gonzalez E., Ferrante J., Van Durme D., Krischer J. (2000). Effects of health insurance and race on colorectal cancer treatments and outcomes. Am. J. Public Health.

[10] Kowal M., Douglas F., Jayne D., Meads D. (2022). Patient choice in colorectal cancer treatment–A systematic review and narrative synthesis of attribute-based stated preference studies. Color. Dis..

[11] Hoehn R.S., Rieser C.J., Phelos H., Sabik L.M., Nassour I., Paniccia A., Zureikat A.H., Tohme S.T. (2021). Association between Medicaid expansion and diagnosis and management of colon cancer. J. Am. Coll. Surg..

[12] Privacy Policy|KFF. https://www.kff.org/privacy-policy/.

[13] Simpson A. How Lack of Medicaid Expansion Fuels Rural Poverty in the Deep South—Center for Public Integrity. https://publicintegrity.org/inequality-poverty-opportunity/medicaid-expansion-in-louisiana-mississippi/.

[14] USCS Data Visualizations—CDC. https://gis.cdc.gov/cancer/USCS/#/AtAGlance/.

[15] Danos D., Leonardi C., Wu X.-C. (2022). Geographic determinants of colorectal cancer in Louisiana. Cancer Causes Control.

[16] Chen V.W., Correa C.N., A Andrews P., Wu X.C., Lucas H.F., Ahmed M.N., A Schmidt B., Rainey J.M. (1999). Louisiana Tumor Registry: New developments and activities. J. State Med. Soc..

[17] ICD-O-3 Site Codes|SEER Training. https://training.seer.cancer.gov/colorectal/abstract-code-stage/codes.html.

[18] Keys A., Fidanza F., Karvonen M.J., Kimura N., Taylor H.L. (1972). Indices of relative weight and obesity. J. Chronic Dis..

[19] Yost K., Perkins C., Cohen R., Morris C., Wright W. (2001). Socioeconomic status and breast cancer incidence in California for different race/ethnic groups. Cancer Causes Control.

[20] U.S. Census Bureau QuickFacts: Louisiana. https://www.census.gov/quickfacts/geo/chart/LA/PST045223.

[21] Gan T., Sinner H.F., Walling S.C., Chen Q., Huang B., Tucker T.C., Patel J.A., Evers B.M., Bhakta A.S. (2019). Impact of the Affordable Care Act on colorectal cancer screening, incidence, and survival in Kentucky. J. Am. Coll. Surg..

[22] Qian Z., Chen X., Pucheril D., Al Khatib K., Lucas M., Nguyen D.-D., McNabb-Baltar J., Lipsitz S.R., Melnitchouk N., Cole A.P. (2023). Long-Term Impact of Medicaid Expansion on Colorectal Cancer Screening in Its Targeted Population. Dig. Dis. Sci..

[23] Freyder L. (2020). Louisiana Behavior Risk Factor Surveillance System. https://ldh.la.gov/page/behavioral-risk-factor-surveillance-system.

[24] Miller S., Wherry L.R. (2017). Health and access to care during the first 2 years of the ACA Medicaid expansions. N. Engl. J. Med..

[25] Mazurenko O., Balio C.P., Agarwal R., Carroll A.E., Menachemi N. (2018). The effects of Medicaid expansion under the ACA: A systematic review. Health Aff..

[26] Yang W., Williams J.H., Hogan P.F., Bruinooge S.S., Rodriguez G.I., Kosty M.P., Bajorin D.F., Hanley A., Muchow A., McMillan N. (2014). Projected supply of and demand for oncologists and radiation oncologists through 2025: An aging, better-insured population will result in shortage. J. Oncol. Pract..

[27] Takvorian S.U., Oganisian A., Mamtani R., Mitra N., Shulman L.N., Bekelman J.E., Werner R.M. (2020). Association of Medicaid expansion under the Affordable Care Act with insurance status, cancer stage, and timely treatment among patients with breast, colon, and lung cancer. JAMA Netw. Open.

[28] Turke P.W. (2021). Five reasons COVID-19 is less severe in younger age-groups. Evol. Med. Public. Health.

[29] Spiers G.F., Kunonga T.P., Hall A., Beyer F., Boulton E., Parker S., Bower P., Craig D., Todd C., Hanratty B. (2021). Measuring frailty in younger populations: A rapid review of evidence. BMJ Open.

[30] Ji X., Shi K.S., Mertens A.C., Zhao J., Yabroff K.R., Castellino S.M., Han X. (2023). Survival in young adults with cancer is associated with Medicaid expansion through the Affordable Care Act. J. Clin. Oncol..

[31] Qian A., Nalawade V., Murphy J.D. (2022). The impact of Medicaid expansion on colorectal cancer incidence among vulnerable populations. J. Clin. Oncol..

[32] Fedewa S.A., Yabroff K.R., Smith R.A., Sauer A.G., Han X., Jemal A. (2019). Changes in breast and colorectal cancer screening after Medicaid expansion under the Affordable Care Act. Am. J. Prev. Med..

[33] Parikh P.B., Yang J., Leigh S., Dorjee K., Parikh R., Sakellarios N., Meng H., Brown D.L. (2014). The impact of financial barriers on access to care, quality of care and vascular morbidity among patients with diabetes and coronary heart disease. J. Gen. Intern. Med..

[34] Bourgeois A., Horrill T.C., Mollison A., Lambert L.K., Stajduhar K.I. (2023). Barriers to cancer treatment and care for people experiencing structural vulnerability: A secondary analysis of ethnographic data. Int. J. Equity Health.

[35] Wolfe M.K., McDonald N.C., Holmes G.M. (2020). Transportation barriers to health care in the United States: Findings from the national health interview survey, 1997–2017. Am. J. Public Health.

[36] Al Shamsi H., Almutairi A.G., Al Mashrafi S., Al Kalbani T. (2020). Implications of language barriers for healthcare: A systematic review. Oman Med. J..

[37] Holgash K., Heberlein M. (2019). Physician Acceptance of New Medicaid Patients: What Matters and What Doesn’t. https://www.healthaffairs.org/content/forefront/physician-acceptance-new-medicaid-patients-matters-and-doesn-t.

